# A Multi-Country Community of Practice to Strengthen Quality Improvement in Low- and Middle-Income Countries: A Quality Improvement Program Description

**DOI:** 10.3390/healthcare14111545

**Published:** 2026-06-02

**Authors:** Samhita Bhargava, Heather A. Haq, Brodus A. Franklin, Elizabeth Davis, Florence Anabwani-Richter, Thobile Bhembe, Lindokuhle P. Dlamini, Makhosazana Dlamini, Andy Chapola, Nomsa Kafumba, Chisomo Mzandu Zinyemba, Menard Bvumbwe, Kyakuwa Richard Jjuuko, Jacqueline Balungi Kanywa, Dithan Kiragga, Andreas Boy Isaac, Esther Makhalanyane, Lwamba Nyembo, Retselisitoe Mahlaha, John T. Farirai, Eunice W. Ketang’enyi, Andrea E. M. Imsen, Iuliana Costas, Susan B. Torrey

**Affiliations:** 1 Department of Pediatrics, Baylor College of Medicine, Houston, TX 77030, USA; 2 Department of Pediatrics, Contra Costa Health, Martinez, CA 94553, USA; 3 Global Health, Texas Children’s Hospital, Houston, TX 77030, USA; 4 Baylor College of Medicine Children’s Foundation-Eswatini, Mbabane, Eswatini; 5 Baylor College of Medicine Children’s Foundation-Malawi, Lilongwe, Malawi; 6 Baylor College of Medicine Children’s Foundation-Uganda, Kampala, Uganda; 7 Baylor College of Medicine Children’s Foundation-Lesotho, Maseru, Lesotho; 8 Botswana-Baylor Children’s Clinical Centre of Excellence Trust, Gaborone, Botswana; 9 Baylor College of Medicine Children’s Foundation-Tanzania, Mbeya, Tanzania; 10 Fundación Baylor Argentina, Añelo Q8305, Neuquén Province, Argentina; 11 Baylor Black Sea Foundation, 900068 Constanța, Romania

**Keywords:** quality improvement, community of practice, global health, low- and middle-income countries, health systems strengthening, capacity building, virtual learning platforms

## Abstract

**Highlights:**

A virtual, multi-country Quality Improvement Community of Practice, using low-cost methods to drive collaboration and learning, is a feasible strategy for strengthening health workforce capacity and engagement in quality improvement activities in low-resource settings.

**What are the main findings?**

- The study demonstrates the feasibility of a virtual, multi-country Quality Improvement Community of Practice (QICoP), which successfully engaged healthcare workers across low- and middle-income countries (LMICs).
- QICoP participation increased by 74%, 90% of network sites were active, and 83% of participants reported improved learning and collaboration.
- Simple, low-cost methods such as WhatsApp reminders and session feedback, effectively increase participation and may offer replicable approaches for other global health initiatives.
- Development of a QI Basics curriculum and the empowerment of local champions, supporting sustainable, site-led quality improvement efforts strengthened capacity for QI program development across the Network.

**What are the implications of the main findings?**

- A virtual community of practice (CoP) supported by simple tools and local champions may be a scalable, cost-effective strategy to strengthen health workforce capacity, enhance program ownership, and support quality improvement programming in resource-limited settings. This approach aligns with national health priorities to build and sustain high quality health systems.

**Abstract:**

Background/Objectives: Quality improvement (QI) is widely used in global health to improve patient outcomes, reduce costs, and strengthen service delivery. The Texas Children’s Global Health Network (TCGHN) includes nine independent non-governmental organizations supporting healthcare in low- and middle-income countries (LMICs), with pediatric HIV clinical centers of excellence in six countries in sub-Saharan Africa (SSA), supported technically by Baylor College of Medicine. We describe the development of a virtual QI Community of Practice (QICoP) to connect geographically dispersed teams and strengthen local QI capacity. Methods: In 2022, QI and global health experts convened to design the QICoP and assess site readiness. Participants were recruited from the sites based on their interest. Meetings were held via Zoom, with attendance, evaluations, and organizer notes tracked. QI tools were used to identify site strengths, challenges, and strategies to improve engagement. Results: From January 2023 to September 2024, the QICoP held 15 sessions, including 3 abstract-writing workshops, averaging 35 participants per session. QI abstract submissions to the annual Network meeting doubled from 2023 to 2024. Across 15 sessions, 83% of participants reported positive experiences. Based on participant feedback and QI sessions from the 2022–2024 Network meetings, we developed a blended QI basics curriculum, recruited site champions to improve communication, and launched a WhatsApp platform to enhance engagement. Conclusions: A virtual QICoP may be a feasible model to support professional development, increase knowledge and idea sharing, and connect individuals across geographies over a shared mission to improve healthcare quality in LMICs.

## 1. Introduction

High-quality health systems are a bedrock for positive health outcomes and socioeconomic progress. Healthcare access in low- and middle-income countries (LMICs) has expanded over the past several decades. However, data suggest that there continue to be significant gaps in the quality of care provided in LMICs, with over 8 million individuals dying annually due to poor quality of care [1]. The United Nations Sustainable Development Goals (SDGs) emphasize the need to provide “access to quality essential healthcare services” across a range of conditions [2]. As countries work toward integrating a quality strategy into their health systems, success will depend on the capacity of existing healthcare workers to provide quality care and become quality advocates at a grassroots level.

An innovative model for integrating capacity-strengthening initiatives into the workplace is the *Community of Practice (CoP)*. A CoP is defined as a “persistent, sustaining social network of individuals who share and develop an overlapping knowledge base, set of beliefs, values, history and experiences focused on a common practice and/or mutual enterprise” [3]. A CoP is defined by three key characteristics: domain, community, and practice [4]. “Domain” refers to the idea of a shared mission. “Community” refers to the social makeup of the group within which capacitation is taking place. It is built on a foundation of inclusivity, trust, and mutual respect. “Practice” refers to the process of developing a new knowledge base or skill set through experiential learning, mentorship, and reflection [4,5]. While CoPs were initially described in business and management, they have also been adapted to healthcare settings where they can serve as a platform for professional development and networking, knowledge sharing, and ultimately improved clinical care. Both in-person and virtual communities of practice have been utilized. Virtual CoPs are a convenient way to connect a geographically dispersed group of professionals and increase access to participation by addressing potential barriers such as cost, transportation, and lack of time. Although data addressing other CoP models are limited, existing evidence suggests that online educational initiatives can be valuable to those living in LMICs [6,7].

The Texas Children’s Global Health Network (TCGHN) comprises nine affiliated, independent non-governmental organizations (NGOs) that partner with the Ministries of Health to provide care for over 365,000 individuals in Argentina, Botswana, Colombia, Eswatini, Lesotho, Malawi, Romania, Tanzania, and Uganda. The NGOs were initially founded as outpatient pediatric HIV treatment centers, known as the Centers of Excellence (CoE). They have since expanded their missions to incorporate maternal/newborn health, inpatient care, public health programming, and satellite CoEs in remote regions. At the time of initiation, over 2400 staff support healthcare delivery across the TCGHN, with ongoing technical support provided by Baylor College of Medicine and Texas Children’s Hospital in Houston, Texas, USA. Previously, TCGHN only offered discrete, in-person Quality Improvement (QI) workshops on an intermittent basis to strengthen Network sites’ capacity to engage in QI activities. These workshops focused more on developing QI projects than cohesive QI program development, and there was no ongoing support for QI programming across the TCGHN. Our goal was to create a virtual Quality Improvement Community of Practice (QICoP) model to support TCGHN-wide QI program development. With this program description, we report our experience to date in implementing a virtual QICoP over a geographically disperse area. Outcomes are descriptive and intended to demonstrate feasibility, acceptability, and early signals of value, rather than generalizable or causal effects.

## 2. Materials and Methods

### 2.1. Approach

We describe the development and early outcomes of an educational program using descriptive programmatic metrics.

### 2.2. Program Development and Participant Recruitment

**Figure 1:**
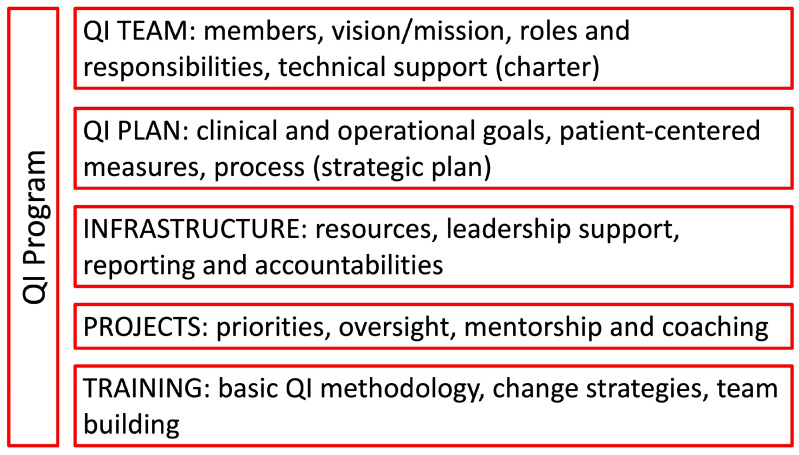
Core components of a Quality Improvement (QI) program framework.

The QICoP model was designed to leverage our prior experience with virtual learning platforms (VLPs) to promote communication between group members across sites and manage membership [6]. We utilized an iterative process to continuously evaluate and modify the model based on feedback after each QICoP meeting and during the TCGHN 2022, 2023, and 2024 Network Meeting QI sessions. At the 2022 Network Meeting, a team of TCGHN QI and global health experts developed a QI strategic plan for the Network. We sought to establish a forum for QI education, exchange of ideas, peer collaboration, and longitudinal engagement to support QI program development utilizing the CoP model and the VLP (Figure 1).

The QICoP concept was launched in 2022 at the annual TCGHN Meeting in Johannesburg, South Africa. Prior to the meeting, we sought feedback and buy-in from the Executive Director of each Network site and developed and distributed a survey to gather baseline information about QI programming from the sites. At the meeting, we conducted a QI program development workshop to explore the readiness for building a QI program, as well as strengths/gaps of current QI processes. Tools that were used to examine the current state of QI initiatives included the Partners in Health Mentorship and Enhanced Supervision for Healthcare and Quality Improvement [8,9]. Participants completed a QI readiness tool we developed based on PRISM, the Practical, Robust Implementation and Sustainability Model [10]. The USAID guiding principles for QI programs were utilized to establish a foundational framework for initiating QI initiatives [11]. Groups from each participating site identified action items related to QI to take back to their workplace. Modifications were made to the model in August 2022 and at subsequent Network Meetings using QI methodology.

Network staff were invited to join the nascent TCGHN QICoP regardless of whether they had an existing in-country QI program. Participants represented various disciplines including medicine, nursing, data, pharmacy, social work, information technology, and nutrition. There were no exclusion criteria. Participation in the QICoP was voluntary. The program evaluation of the QICoP did not constitute human subjects research as per the Baylor College of Medicine Institutional Review Board and therefore was exempt from review.

### 2.3. Program Structure and Content

Monthly QICoP sessions lasting approximately 1.5 h were conducted over Zoom beginning in January 2023. Each session was planned and facilitated by the QICoP support team in Houston, with emphasis on facilitating discussion amongst QICoP members. Core discussion topics included support to develop terms of reference for local QI teams, exploring the current state of a problem using QI tools, use of data to track clinical performance, an introduction to plan-do-study-act cycles, establishment of a QI team, and QI program development. Participants were encouraged to share ideas and ask questions via the chat function or verbally during the meetings. QICoP sessions were recorded to allow access on the VLP. Special workshops to discuss QI abstract-writing and QI oral/poster presentations were held annually in preparation for the Network Meeting. A new, dedicated QI abstract category was created for the November 2023 Network meeting and coaching was offered to support authors submitting QI abstracts.

In the second year, virtual Network-wide meetings were held on an alternating month basis. Site-specific QICoP meetings were initiated on alternating months to provide individually tailored support to each QI program. QI site champions were recruited at each site to serve as communication liaisons between the local QI team and the QICoP support team in Houston. In addition, live Spanish interpretation services were incorporated into each QICoP meeting to facilitate participation of sites based in South America.

### 2.4. Session and Program Evaluation and Data Analysis

Session evaluations: For each QICoP session and Network Meeting QI workshop, attendance and facilitator notes were recorded. Participant perceptions and engagement metrics were prioritized as primary outcomes given their central role in feasibility and sustainability of educational innovations. We created a standard participant feedback survey that was not formally validated. It was administered using SurveyMonkey (Momentive, San Mateo, CA, USA). Surveys were anonymous to maintain confidentiality. Each session evaluation included 5-point Likert scale and open-ended questions. Standard descriptive statistics were used to analyze participation and quantitative survey response data. We informally reviewed open-ended responses to identify common themes.

Program evaluation: During the QICoP session in August 2023 we used QI tools to evaluate the effectiveness of the QICoP to date. With fishbone and key driver diagrams, we explored factors contributing to QICoP engagement and process strength. The QICoP convened in-person at the 2023 Network meeting in Johannesburg, South Africa to further evaluate our experience and develop a strategic plan for 2024 for both the QICoP and each local QI program.

## 3. Results

Readiness for site-level QI program development was assessed at the 2022 TCGHN Meeting. Seven of 9 sites participated. Colleagues from Colombia and Romania were not present. There was heterogeneity regarding resources and experience with QI program development across Network sites, with some sites having more mature QI programs and other sites without any active formal QI infrastructure.

Common themes included the following:

- **Organizational challenges:** Alignment with strategic priorities is good. Lack of dedicated financial and human resources to support QI program development is a challenge. Recognizing and addressing culture in the workplace is important.
- **Leadership engagement:** Executive Director-level support for QI was present across the Network. All sites had a QI program with varying degrees of organization, although some sites’ teams were inactive. All sites had staff with limited experience and knowledge about QI methodology; however, individuals with extensive QI experience and knowledge at some sites were also identified.
- **Access to data:** Data are available across Network sites with an electronic medical record. Some sites also have data available through national databases. Monitoring and evaluation (M&E) support is generally available, though sites had varying degrees of routine data review by clinical and program staff. Several sites have M&E representatives on the QI team.

The virtual QICoP officially launched in January 2023. Although Colombia and Romania were not present at the 2022 TCGHN meeting, representatives from both countries were included in the launch. Over a span of nearly two years, from January 2023 to September 2024, the QICoP held a total of 15 sessions, including 3 workshops to support QI abstract writing, with an average of 35 participants per session. Participants included nurses, clinical officers, medical officers, medical directors, and other support staff. QI abstract submissions to the annual Network Meeting increased from 7 in 2022 (prior to initiation of the QICoP) to 13 in 2023, and 27 in 2024.

### 3.1. Session Evaluations

Quantitative survey responses from participants across 15 QI sessions included information about learning environment; effectiveness of sessions for improving knowledge, skills and attitudes; opportunities for collaboration among participants; success at creating a sense of community; whether personal expectations were met; and presence of internet or other technology issues. On average, 83% of responses were positive, although with an average response rate of about 30%, this should be considered when interpreting the proportion of positive responses. Eleven percent were not positive with the remaining undecided (Table 1). Table 1 includes a sample of qualitative responses.

### 3.2. Program Evaluation and Intervention

At session 7 (August 2023) of the QICoP, participants identified the following opportunities for improvement: poor engagement in QICoP sessions, a better understanding of local strengths and challenges, and limited knowledge of QI methodology. As an educational exercise, the QICoP members used QI methodology to design and implement the following onetime intervention to address them.

### 3.3. Poor Engagement

**Figure 2:**
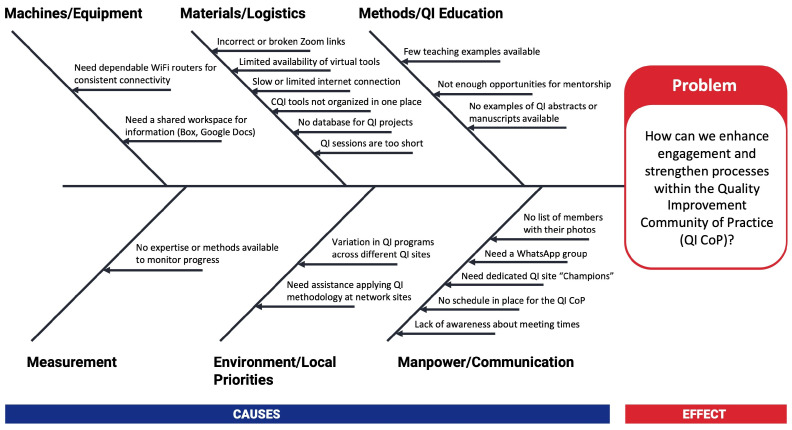
Fishbone diagram highlighting strategies to strengthen engagement in the QI Community of Practice (QICoP).

Using a Fishbone diagram (Figure 2), participants categorized underlying issues affecting participation. Issues regarding technological infrastructure included slow or unreliable internet connections, incorrect or broken Zoom links, limited availability of virtual tools, and lack of dependable Wi-Fi routers. Other concerns included availability of virtual resources and QI databases, limited QI knowledge, access to virtual platforms to enhance cross-site collaboration, and variation in QI programs between sites. Communication challenges, competing local priorities, and opportunities for mentorship and teaching were also significant concerns.

**Figure 3:**
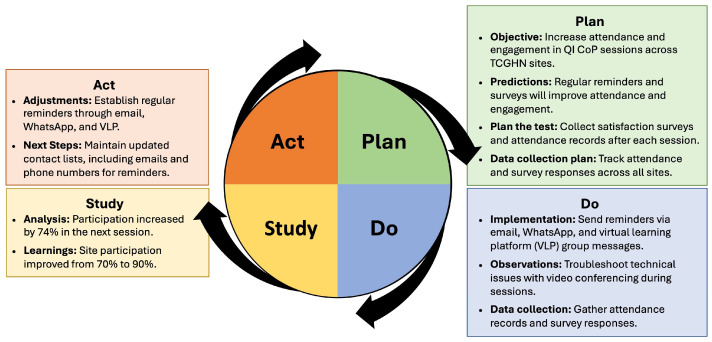
Plan-Do-Study-Act (PDSA) cycle to improve attendance and engagement in the QI Community of Practice (QICoP).

**Figure 4:**
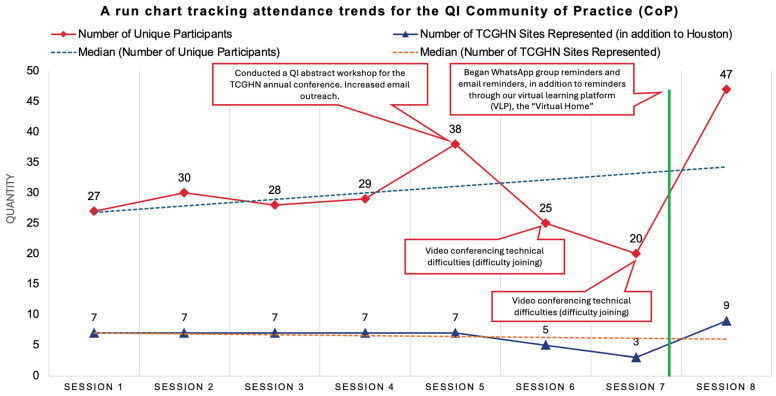
Overall trends in attendance and site participation in the QI Community of Practice (QICoP).

Based on these insights and available resources, we prioritized communication-based drivers for our first project. We used the Plan-Do-Study-Act (PDSA) quality improvement methodology to address these communication-based barriers to engagement in the QICoP. The interventions included creating a WhatsApp group, sending regular reminders prior to each QICoP session, and evaluating satisfaction following each session. Data to measure effectiveness included attendance records and satisfaction survey responses. During implementation prior to session 8, reminders were disseminated through email, WhatsApp, and VLP group messages prior to each session. Analysis of data collected during the only PDSA cycle (Figure 3) demonstrated an increase in participation, with attendance rising 74% in the session immediately following the introduction of the reminders and site participation improving from 70% to 90%. However, this improvement was not consistently sustained. Subsequent sessions demonstrated variability and decline in both attendance and site participation. Project results are summarized in a run chart that includes data before and after implementation of the interventions (Figure 4). These observations informed ongoing adaptations to the program, including continued use of communication strategies and exploration of additional approaches to support sustained engagement. The QICoP continued to evolve in response to these observations. Adjustments were made to ensure the regularity of reminders through established communication channels. Maintaining up-to-date contact lists, including email addresses and phone numbers, was identified as a key step for sustaining these improvements moving forward. However, we cannot comment on the effectiveness of these efforts since we did not analyze subsequent data.

### 3.4. Local Strengths and Challenges

**Figure 5:**
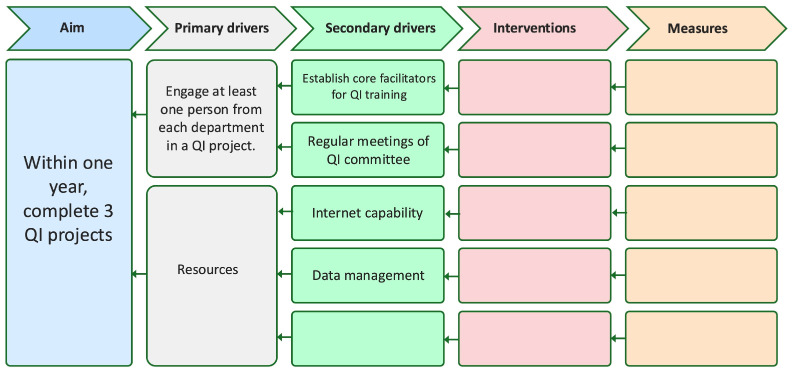
Sample key driver template for local strengths and challenges. Interventions and measures columns intentionally left blank for site completion.

We developed and conducted a workshop at a meeting of the QICoP during the 2023 TCGHN Meeting to better understand local strengths and challenges. Participants used a key driver diagram to identify drivers of strengths and challenges at their site, with the aim of improving a specific aspect of QI programming at their sites during the following year. See a sample template below (Figure 5). Consistent themes emerged across all sites. Strengths included management support and enthusiastic QI teams. Staff availability, competing priorities, documentation, and QI knowledge were common challenges. Key drivers included staff, data, time, and management support. Visions for 2024 included support for QI teams, developing a culture of QI across the organization, and initiating more client driven projects.

### 3.5. Quality Improvement (QI) Methodology Knowledge: QI Basics Curriculum

To address the identified QI methodology gaps across sites, a member of the QI support team in Houston created a QI Basics curriculum tailored to the clinical settings and needs of the TCGHN. This blended learning course included six self-directed didactic lessons to be completed asynchronously and two synchronous cross-Network learning sessions. The intent for the course was for local teams to use it to strengthen their understanding of QI methodology. Lesson 1 included an overview of QI and introduction to the Model for Improvement, while Lessons 2–6 reviewed each step in the Model for Improvement in detail. A sample case was introduced in Lesson 1 and expanded upon with each subsequent lesson. The QI Basics course was piloted in July 2024 using a train-the-trainer model. QICoP site champions recruited 12–14 participants from each site. The QI Basics pilot participants were given two weeks to review each lesson either independently or with their site colleagues and provide feedback through a survey after each lesson block. Office hours were offered via Zoom after each 2-week block to clarify any questions and address concerns. A 1.5 h synchronous virtual session was conducted using Zoom after Lessons 1–3 and again after Lessons 4–6 to review the content covered. During the synchronous virtual sessions, a breakout activity facilitated by QI experts identified by the Houston QI support team, offered participants the opportunity to build a hypothetical QI project based on the tools introduced in the QI Basics course. At the 2024 TCGHN Meeting, a subset of QI Basics pilot participants received training and practice on teaching QI Basics. Following this train-the-trainer session at the Network Meeting, participants returned home to develop a plan for teaching QI Basics to other site staff members.

## 4. Discussion

This program description described development and feasibility of an interdisciplinary, multi-country QICoP within the TCGHN. The QICoP model was conceptualized in October 2022, followed by an assessment of QI readiness at Network sites in November 2022. Participants were recruited from local sites based on their interest in QI. The virtual QICoP launched in January 2023, and in July 2024, the QI Basics Pilot Course was introduced to bridge knowledge gaps in quality improvement methodology. Utilizing the PDSA quality improvement methodology with an emphasis on communication-based interventions, we observed: (1) attendance increased by 74% following the implementation of targeted strategies, (2) 83% of participants reported enhanced learning and collaboration, (3) 90% of Network sites actively engaged in QICoP sessions, and (4) the number of QI abstract submissions to the Network Meeting almost quadrupled, underscoring the potential of virtual CoPs as one component in fostering quality improvement initiatives across LMICs. These trends likely reflect multiple contributing factors, including abstract-writing workshops, coaching support, and the introduction of a dedicated QI abstract category at the Network Meeting, alongside the QICoP.

Similar to other CoP models for capacity building [12], the QICoP may be a promising model to support the domain of QI program development across our geographically dispersed network. The QICoP linked professionals from the TCGHN sites in Lesotho, Tanzania, Malawi, Eswatini, Uganda, Botswana, Romania, Argentina, Colombia, and the QICoP support team in Houston. Prior to the formation of the QICoP in 2023, there was variable involvement in QI work both at the program and project levels at each site. The initiation of monthly QICoP meetings has created a community through which participants can develop their QI skills and programs together. The reception of the CoP by the participants has generally been positive as indicated by participation in CoP sessions and survey responses. Our experience is consistent with that of other CoPs that have been created as capacity-strengthening initiatives in the global health space [6,7,13].

Our work in creating a QICoP has been an iterative process that focuses on building an inclusive, supportive community that works together to enhance quality improvement efforts across the Network. During the first year, we identified effective communication as a challenge to participants. Using elements from the Model for Improvement, we initiated email and WhatsApp groups to ensure that members were informed about meeting times and CoP activities. In the second year, we arranged for a live, simultaneous Spanish interpreter to be present at each meeting, allowing interested participants from the Spanish-speaking countries in the Network to become involved in the CoP activities.

Group learning and mentoring are essential characteristics of successful communities of practice. In the initial phase of the QICoP, discussions were primarily led by the Houston support team, with participant contributions remaining sporadic and infrequent. In addition, several factors made it difficult to assess member engagement during the sessions. Almost all members had turned off their cameras due to Internet bandwidth issues. Participants would often share a single computer and assign a spokesperson, potentially limiting the contributions of other members in the room to the conversation. In 2024, based on participant feedback, we recruited a volunteer champion from each site to serve as the liaison between the QICoP support in Houston and their local colleagues. The site champion role thus far has involved sending reminders regarding upcoming QICoP meetings, improving logistics for accessing the Zoom link, providing site updates at the beginning of each meeting, working with the Houston support team to organize site-specific meetings, and disseminating information regarding the QI Basics pilot course to site participants. The QI site champions provide continuity to participants who may not be able to attend sessions due to competing priorities. They have been instrumental in communicating strengths/challenges of their site QI program and in raising awareness and enthusiasm for QI among their colleagues. We learned that adaptability of a CoP based on participant feedback and priorities is an important characteristic in maintaining commitment to the group, as has also been highlighted by Fruchtman et al. [14].

Variable degrees of QI knowledge and experience have also impacted members’ ability to fully participate in session activities. To address these gaps, QICoP session content has included a review of QI tools and breakout activities that offer exploration of the tools in a more intimate setting. We are also expanding dedicated QI training to include developing more basic educational material as well as supporting local training initiatives. Preliminary experiences suggest that consistent with the findings of Nguyen and colleagues, the QICoP abstract writing workshops contributed to enhanced scholarly output [7]. In order to support QI program/project development at the site level and provide individualized mentorship, the Houston support team regularly meets with local QI teams. As a result of these sessions, the Houston support team has been able to incorporate more direct participation from sites in developing agendas and activities for QICoP sessions, reinforcing the observation made by Fruchtman et al. that specific roles and responsibilities increase engagement in a CoP.

While early outcomes of the QICoP have been encouraging, there are several limitations to this model of social learning in our context. First, given the retrospective nature of this evaluation, we must consider contributing biases and sub-optimal data quality. Participant recruitment was voluntary. The survey response rate was modest, and the accuracy of attendance data has been variable. The QI CoP sessions were conducted in English. This may have limited participation from our sites in Colombia and Romania. In addition, although English is a national language at the TCGHN sites in sub-Saharan Africa, QICoP team members may be more comfortable conversing and learning in their native language. The clinical programming and contexts at our sites in sub-Saharan Africa are similar, in contrast to the sites in Colombia and Romania. This may have affected the relevance of our programming for those sites. It is challenging to establish trusted relationships, particularly in a multidisciplinary and multicultural group, on a virtual platform. Other virtual CoPs have experienced similar challenges [13,14,15]. Familiarity with technology and reliable Internet access are also well-recognized barriers to engagement on a virtual platform. Finally, access to onsite mentorship opportunities is also a challenge. While we have incorporated site-specific check-ins into the QICoP meeting schedule, access to local QI expertise varies by site and may not be consistent. Without the guidance of an individual with experience in improvement science, novice QI teams may struggle to commence and sustain their QI work. While outcome measures focused primarily on participant engagement and perceptions, these indicators were intentionally selected as core measures of feasibility, acceptability, and sustainability for this early-stage educational initiative. As the program matures, future work may explore longitudinal and system-level outcomes to extend these preliminary programmatic findings.

This study describes our initial experiences in an actively evolving virtual CoP. As we continue to expand our QICoP, it is important to consider the typology of virtual CoP learning (co)evolution described by Eller et al. [16]. To date, our sessions have been facilitated primarily by the Houston-based support team of QI experts. Moving forward, we aim to cultivate a dynamic in which members themselves lead sessions, and meaningful knowledge exchange and networking take place before, during, and after formal QICoP meetings. Members could choose to undergo additional training in QI science to become “master trainers” and support local QI initiatives. While the early focus of the QICoP has been on QI education, as the members gain experience in QI methodology, the forum can also be used as a space to present QI projects. In alignment with the recommendations of Eller et al. [16], we are developing a centralized repository of resources such as a website, online courses, discussion boards, case studies, and past/ongoing QI projects to further support the quality improvement efforts of our members. An evolution toward a member-led CoP would align with the goal of fostering more horizontal collaboration between high-income countries (HICs) and LMICs within the TCGHN [16]. Supporting local and cross-site CoP and QI initiatives may also promote a bottom-up approach to Network-wide CoP efforts, by creating opportunities to apply QI theory in practice and enabling peer accountability toward shared improvement goals [14]. As local QI programs mature, the QICoP will need to support local QI programs to address challenges in service delivery. These include lack of a dedicated budget and protected time for QI program development, low motivation/agency to change existing practices, and competing priorities set by leadership.

## 5. Conclusions

A virtual QICoP may be a promising low-cost model to support QI program development and offers opportunities for continuing education, networking, resource-sharing, and innovation across diverse geographical locations. It could be generalizable if these results can be replicated using a more rigorous approach.

## Figures and Tables

**Table 1:** Quality improvement series session qualitative participant survey responses.

Select Themes	Representative Quotations
Open discussion	“Clear presentation…well-structured and open discussions provided new ways of [improving services].” (July 2024) “Open interaction.” (March 2024)
Learning from others	“I love the sharing and hearing from other sites” (July 2024) “Getting to hear what other sites are doing to enhance care with QI projects made me also think that we could actually measure the work we are doing” (July 2024) “Learned about the ‘rich’ expertise in the QICoP Team” (Jan 2024) “Learned that sustainability becomes a major challenge.” (March 2024) “Learning about [QI project] tracking tool from Eswatini team.” (March 2024)
QI education	“[Gained] more knowledge on how to build QI team, [implement] QI activities and [got] feedback on ongoing QI projects.” (Jan 2024)
Mentorship	“Engagement of local QI mentors is really a brilliant idea. I believe it would help build up all QI efforts locally across all the Foundations.” “[Received] support with QI abstracts.”
Areas for change	“Have regional CoP sessions in between the main CoP sessions and brainstorm ideas with regional counterparts on improving QI initiatives across the network.” (Jan 2024) “A session about monitoring of QI projects” (April 2024) “Panel discussions on QI for better engagement from all sites” (Jan 2024) “QI project presentations using an agreed template.” (Jan 2024) “Case discussions.” (March 2024)

## Data Availability

Inquiries about original contributions or data can be directed to the corresponding author.

## Abbreviations

The following abbreviations are used in this manuscript:

AIDS	Acquired Immunodeficiency Syndrome
BCM	Baylor College of Medicine
COE	Center of Excellence
CoP	Community of Practice
LMICs	Low- and Middle-Income Countries
M&E	Monitoring and Evaluation
NGOs	Non-Governmental Organizations
PDSA	Plan–Do–Study–Act
PRISM	Practical, Robust Implementation and Sustainability Model
QI	Quality Improvement
QICoP	Quality Improvement Community of Practice
SDGs	Sustainable Development Goals
SSA	Sub-Saharan Africa
TCGHN	Texas Children’s Global Health Network
USA	United States of America
USAID	United States Agency for International Development
VLP	Virtual Learning Platform
